# Repeated Bout Effect of Downhill Running on Physiological Markers of Effort and Post Exercise Perception of Soreness in Trained Female Distance Runners

**DOI:** 10.3390/sports12060169

**Published:** 2024-06-17

**Authors:** Jason Tallis, Caitlin McMorrow, Sharn P. Shelley, Steven J. Eustace

**Affiliations:** 1 Research Centre for Physical Activity, Sport and Exercise Science, Coventry University, Coventry CV1 5FB, UK; ad9173@coventry.ac.uk (S.P.S.); ac8905@coventry.ac.uk (S.J.E.); 2 School of Life Sciences, Coventry University, Coventry CV1 5FB, UK; mcmorroc@uni.coventry.ac.uk

**Keywords:** exercise physiology, eccentric exercise, delayed onset of muscle soreness, endurance, running

## Abstract

This study examined the effect of repeated bouts of level and downhill running on physiological markers of effort and exercise-induced muscle soreness in trained female distance runners. Ten participants (Age: 24.4 ± 2.0 years; V̇O_2peak_: 52.9 ± 1.1 mL·kg^−1^·min^−1^), naïve to downhill running, completed six alternate 5 min trials of level and downhill running (−15%) at a 70% velocity at V̇O_2peak_ on two occasions, three weeks apart. Perceived muscle soreness was measured upon completion and in the 72 h post exercise. V̇O_2_, Heart Rate (HR), Blood Lactate (BLa), and Respiratory Exchange Ratio (RER) were lower running downhill (*p* < 0.016, ηp^2^ > 0.541). For the first downhill run, Rating of Perceived Exertion (RPE) was higher compared to that for level running (*p* = 0.051; *d =* 0.447), but for the remaining trials, RPE was lower when running downhill (*p* < 0.004; *d >* 0.745). V̇O_2_, HR, and RER were not different in the second bout (*p* > 0.070, ηp^2^ < 0.318); however, V̇O_2_ was lower in each downhill trial (Δ = 1.6–2.2 mL·kg^−1^·min^−1^; *d* = 0.382–0.426). In the second bout, BLa was lower (*p* = 0.005, ηp^2^ = 0.602), RPE in the first trial was lower (*p* = 0.002; *d =* 0.923), and post exercise perceived soreness of the gastrocnemius, quadriceps, and hamstrings was attenuated (*p* < 0.002; ηp^2^ > 0.693). Perceived soreness of the gluteal muscles was lower in the second bout immediately post exercise, 24 h, and 48 h post exercise (*p* < 0.025; *d >* 0.922). A repeated bout of downhill running attenuated perceived muscle soreness and may modulate the physiological and perceived physical demand of a second bout of level and downhill running.

## 1. Introduction

Downhill running has been used as a model to explore the physiological consequences of eccentric muscle activity and the effects of exercise-induced muscle damage [1]. Running at a negative gradient increases the negative velocity of the centre of mass, resulting in altered gait kinematics and increased eccentric action of the lower limb musculature, which aids in controlling deceleration and managing impact transients during the first period of foot ground contact. Given that eccentric force generation physiologically differs from other forms of muscle ctivity [2] and that an unaccustomed increase in eccentric muscle action evokes tissue damage [3], downhill running and its consequences are physiologically and biomechanically distinct.

The acute effects of downhill running on gait biomechanics and running energetics have been thoroughly explored. It is established that downhill running at an equivalent velocity to level running results in reduced V̇O_2_, Respiratory Exchange Ratio (RER), Heart Rate (HR), and metabolic power [4,5,6], and, as such, is less metabolically demanding. Such effects are primarily attributed to an increase in metabolically efficient eccentric muscle actions [7]. Biomechanically, downhill running results in an increased stride frequency, increased aerial time, and decreased duty factor when compared to level running [8], where such effects may contribute to an improved running economy, but also help in reducing the elevated impact transients evoked by downhill running. The reduced metabolic cost of downhill running is not incremental given the increased metabolic demand of energy dissipation [4]. Evidence indicates that, at gradients of −20% and greater, the energetic cost of running begins to increase [8]. Furthermore, due to an increase in unaccustomed eccentric muscle actions, downhill running evokes exercise-induced muscle damage, and the post exercise effects and time course of recovery has been thoroughly explored. Specifically, an initial novel bout of downhill running evokes reduced skeletal muscle contractile function, reduced running economy, an increased perception of pain, and the elevation of several biochemical markers of muscle damage and inflammation [9,10,11,12,13,14], which all persist for days following the downhill running bout.

Unaccustomed eccentric exercise has also been shown to elicit a repeated bout effect (RBE), where skeletal muscle adapts to exercise-induced damage via several protective mechanisms to promote resistance to subsequent muscle-damaging activity [15]. Whilst the time course of effects is not well explored, evidence suggests that protection induced from the initial bout of damaging exercise can persist for up to six months [16]. Although work is ongoing to more precisely elucidate the underlying mechanisms, evidence suggests that the RBE can manifest due to a shift toward the preferential recruitment of low-threshold motor units and improved motor unit synchronisation, altered tendon compliance, remodelling of the skeletal muscle extracellular matrix, and modified inflammatory responses [15], which, mechanistically, may lead to changes in the physiological demand of the activity. Several studies have examined the RBE of downhill running, typically focusing on the impact of the first bout on exercise-induced muscle damage following the subsequent bout. There is consensus in the literature that a repeated bout of the same downhill running protocol attenuates the post-exercise increase in circulating creatine kinase activity, immunoglobulin concentration, and markers of oxidative stress and reduces the magnitude and recovery of losses in maximal voluntary contractile function and the perception of muscle soreness [9,10,11].

Despite a wealth of evidence examining the RBE of downhill running, important knowledge gaps remain. Specifically, given the suggested mechanisms of the RBE, the potential of downhill running to evoke improved running energetics in the subsequent bout has been seldom explored, where current evidence is specific to a single study. Khassetarash et al. [11] examined the effects of repeated bouts of 30 min −20% gradient downhill running on the energetic cost of 5 min level running immediately following the damaging exercise bout. V̇O_2_ and the energetic cost of running were unaffected immediately after the downhill running exercise, whilst V̇E was decreased. V̇O_2_ measured during subsequent 5 min level running bouts 24 and 48 h following the downhill running trial was reduced following the second bout, which was attributed to attenuated muscle damage. Whilst these data offer valuable insights, the current understanding of the effect of the RBE on the physiological demand of running is limited to short-duration level running, and effects on longer-duration activity, crossover effects to downhill running, and the consideration of undulating running terrains are yet to be examined and are explored in the current study. Developing this understanding is important in understanding the conditioning potential of downhill running for evoking an improved distance running performance. Furthermore, previous research evaluating acute responses and the RBE of downhill running is specific to long-duration protocols (typically 30 min or greater [1]), and the impact of shorter-duration downhill running, which may be practically more advantageous for athlete conditioning, is yet to be explored. As such, the present study evaluated the effect of repeated bouts of level and downhill running on physiological markers of effort and exercise-induced muscle soreness in trained female distance runners. It was hypothesised that conditioning evoked by the first bout of exercise would reduce V̇O_2_, RER, HR, BLa, and RPE in the repeated bout of level and downhill running and attenuate post exercise muscle soreness.

## 2. Materials and Methods

To complete this experimental study, participants were asked to attend the human performance laboratory at the host institute on three separate occasions, abstaining from intense physical activity in the 48 h prior. In the first visit, the participants completed a graded incremental exercise test to determine their VO_2peak_ and were familiarised with the experimental protocol. In the remaining two visits, the participants completed the experimental protocol, with each visit separated by three weeks. Prior to each session, the participants were asked to complete a departmental health screen questionnaire. The participants were asked to abstain from strenuous physical activity 72 h following each visit and all experiments were conducted at the same time of day to control for circadian effects.

### 2.1. Participants

Following ethics approval (Ref: P127140) and the completion of informed written consent, trained female distance runners (Tier 2 in accordance with the classification criteria outlined by McKay et al. [17]), with a minimum of two years’ experience, were recruited from the university running club and running clubs local to the host institute. One participant dropped out due to illness and another due to reasons not stated, leaving a total sample of 10. The participants were competitive distance runners, were all apparently healthy (determined by completion of the Physical Activity Readiness Questionnaire), and had been injury-free for a minimum of 6 months. The participants had not previously performed prolonged downhill running, but were familiar with treadmill running at level and positive gradients. Those with experience of downhill running, who were not tier 2 competitive athletes, and who were not injury-free or had a health contradiction preventing safe completion of the physical tasks were excluded. The participant characteristics are reported in Table 1.

### 2.2. Graded Incremental Exercise Test and Familiarisation

Initially height (cm) was measured using a stadiometer (Model 220, Seca, Hamburg, Germany), and body mass (kg) was measured by digital floor standing scales (Model 770, Seca, Hamburg, Germany). Following 10 min of seated rest and a standardised warm-up consisting of 5 min running at 7 km·h^−1^, followed by a series of upper and lower body dynamic stretches, the participants completed a V̇O_2peak_ test, where the participants were asked to compete a ramp test protocol until volitional exhaustion on a motorised treadmill (HP cosmos Saturn, HP Cosmo Sports & Medical GmbH, Nussdorf-Traunstein, Germany). Starting at 6 km·h^−1^, the velocity was increased by 1 km·h^−1^ every 2 min. Throughout the test, expired gas was measured using a Metalyser (Cortex Metalyser 3B, Cortex Biophysik, Leipzig, Germany), which was calibrated in accordance with the manufacturer’s instructions prior to each test. Heart rate (HR) was continually monitored using a chest-fitted telemetric HR monitor (Polar FT1, Kempele, Finland), and Rating of Perceived Exertion (RPE 6–20 scale) was measured using the Borg scale. Within the first minute following the completion of the test, blood lactate (BLa) concentration was determined by means of a finger prick capillary sample. Initially, the finger was wiped with an isopropyl alcohol swab (Medlock Medical, Oldham, UK), punctured using a 1.8 mm lancing device (Safety Lancet, Sarstedt, Germany), and the initial blood was wiped away with a tissue. A 0.7 µL sample was collected and BLa determined using a Lactate Plus Meter (Nova biomedical, Waltham, MA, USA). V̇O_2peak_ was determined as a Respiratory Exchange Ratio (RER) over 1.1, BLa greater than 8 mmol/L^−1^, HR ± 10 beats of predicted maximal (220-age), and RPE greater than 17, where all conditions were met. RER was averaged over the final 60 s of the final stage, and HR and RPE were recorded at volitional exhaustion.

After a period of recovery (~20 min), the participants were asked to complete a 5 min trial of level running followed by a 5 min trial of downhill running at the velocity and gradient to be used in the experimental trial. This served as a familiarisation with the running tasks to be completed in the experimental protocol.

### 2.3. Experimental Protocol

**Figure 1:**
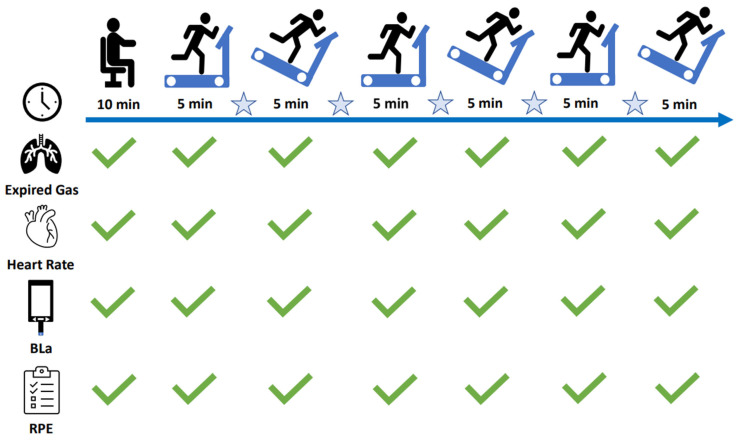
Schematic of experimental protocol. Star = 2-min rest; BLa = Blood lactate; RPE = Rating of Perceived Exertion.

The experimental protocol was completed in accordance with that outlined in Figure 1. Following 10 min of seated rest, BLa, HR, V̇O_2_, and RER evaluated from measurements of expired gas were determined in the manner previously described. Upon completion of the standardised warm-up, the participants completed six alternate 5 min trials of level and downhill running, each separated by 2 min standing passive rest. Previous work typically employed a single-effort 30 min trial of downhill running [1]. Given that such long durations of downhill running cause substantial muscle damage and impaired function, a shorter trial of downhill running was selected for the present study, which may be practically more advantageous for athlete conditioning. Furthermore, this protocol was selected to examine the crossover potential of the RBE on multiple trials of level and downhill running.

In accordance with previous literature examining the physiological and biomechanical effects of downhill running, for downhill trials participants ran at a gradient of −15% [18,19] and at the velocity that represented 70% of the velocity achieved at V̇O_2peak_ [19,20]. Five-minute trials represent the minimum time required to see a plateau in expired gas outcome measures [18]. From expired gas, V̇O_2_ and RER were averaged over the final 60 s of each trial, and HR and RPE were recorded in the final 30 s of each trial. Two-minute rest intervals between trials were used to measure BLa and change the gradient of the treadmill. Upon completion of the test, the participants were allowed to walk or run at a self-selected pace for 5 min.

Immediately upon completion of the treadmill running protocol, the participants were asked to evaluate their regional perception of muscle soreness using a modified version of the Talag [21] 0–6-point scale, anchored at one end with a score of “0—No discomfort” and at the other “6—Unbearable Pain”. Participants completed three sets of 10 squats, and upon completion of each set, completed the perception of pain scale focused separately on the gastrocnemius, quadriceps, hamstrings, and gluteal muscles. An average of the three scores for each muscle/muscle group was recorded. This procedure was repeated 24, 48, and 72 h following, and participants were asked to abstain from intense physical activity during this period. These procedures were repeated in the second experimental trial, which occurred a minimum of 3 weeks later. Evidence suggests that the RBE can last up to 6 months [16], however, the time course of the RBE elicited from downhill running is yet to be explored and there is little consensus in the literature regarding the most suitable time to complete the second bout. Typically, previous work has separated bouts by periods ranging from 5 days to 5 weeks [1]. Given that following eccentric exercise, the restoration of muscle force generating ability can take 7–14 days [15], a 3 week recovery period was afforded between bouts and is in keeping with the timeframe used in previous work that has demonstrated RBE-induced neural and biomechanical changes in gait [11,22]. Assessments took place within the competitive season, however, in the period between bouts participants were asked to abstain from competition but maintain normal training.

### 2.4. Statistical Analysis

Statistical analysis was performed using SPSS v.28 (SPSS Statistics for Windows, IBM Corp., Armonk, NY, USA) and graphical representation using GraphPad Prism v.9 (GraphPad Software, Boston, CA, USA). All data are presented as mean ± standard error of mean (SEM). Shapiro–Wilk and visual inspection of Q-Q plots indicated that the data were approximately normally distributed, justifying the application of parametric statistical tests. In order to evaluate parity in starting conditions, between-bout differences in the resting physiological variables (V̇O_2_, HR, BLa, and RER) were assessed using a paired samples t-test. V̇O_2_, HR, BLa, RER, and RPE measured during the exercise trials were analysed using three-factor repeated measures Analysis of Variance (ANOVA) with Bout (1 vs. 2), Gradient (level vs. downhill), and Trial (1 vs. 2 vs. 3) as factors. Perception of pain was analysed using two-factor repeated measures ANOVA with Bout (1 vs. 2) and Time (immediately post vs. 24 h post vs. 48 h post vs. 72 h post) as factors. Where applicable, Greenhouse–Geisser, recognised as a more conservative epsilon correction, was used to correct the degrees of freedom for the F-distribution on occasions when sphericity was violated. To provide context for the magnitude of any differences found, effect size calculations appropriate to each statistical test were performed. For ANOVA, partial eta squared (ηp^2^) was calculated to estimate effect sizes and were classified as small (<0.05), moderate (0.06–0.137), or large (>0.138) (Cohen, 1988) [23]. Where applicable, significant main effects and interactions were explored using Bonferroni-corrected pairwise comparisons and Cohen’s *d* effect sizes determined, and were interpreted as trivial (<0.2), small (0.2–0.6), moderate (0.6–1.2), or large (>1.2) (Hopkins et al., 2009) [24]. The level of significance was set at *p* ≤ 0.05.

## 3. Results

### 3.1. Physiological Variables

When measured at rest, there was no difference in V̇O_2_ (0.05 ± 0.02 mL·kg^−1^·min^−1^ vs. 0.09 ± 0.03 mL·kg^−1^·min^−1^; *p* = 0.681; *d =* 0.153), RER (0.86 ± 0.01 vs. 0.89 ± 0.14; *p* = 0.138; *d =* 0.641), HR (64.7 ± 0.2.96 bpm^−1^ vs. 68.7 ± 2.75 bpm^−1^; *p* = 0.132; *d =* 0.443), or BLa (1.46 ± 0.08 mmol/L^−1^ vs. 1.26 ± 0.13 mmol/L^−1^; *p* = 0.125; *d =* 0.593) between the first and second bout.

**Figure 2:**
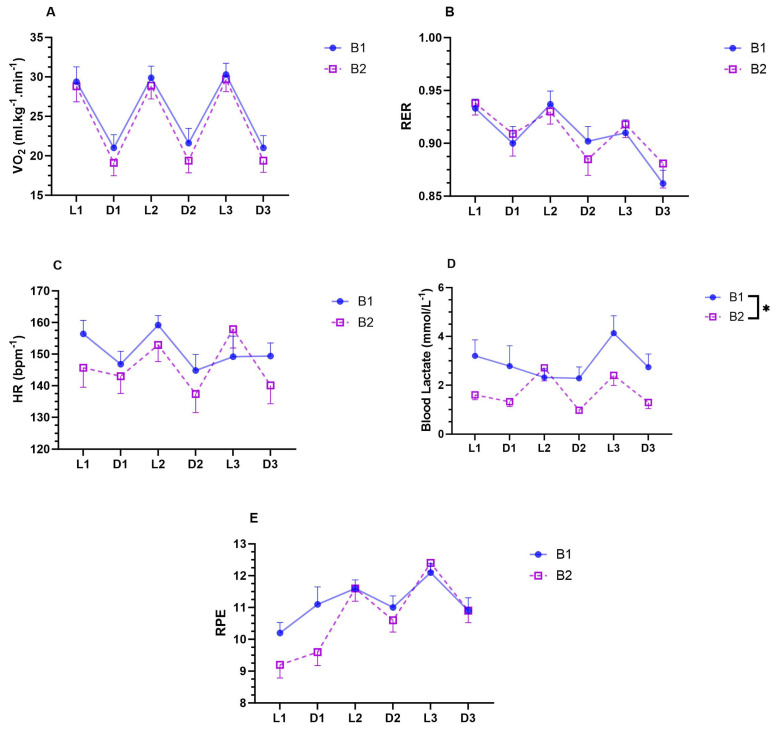
The effect of repeated bouts of downhill and level running on VO_2_ (**A**), RER (**B**), HR (**C**) BLa (**D**), and RPE (**E**). [Data represented as mean ± standard error of mean (SEM); n = 10; L = Level running; D = Downhill Running; B = Bout; * represent *p* < 0.05 for ANOVA main effect of bout].

During the treadmill protocol, there was a main effect of gradient with a lower V̇O_2_ when running downhill compared to level running (Figure 2A. *p* < 0.001, ηp^2^ = 0.973). However, there was no main effect of bout (Figure 2A. *p* = 0.071, ηp^2^ = 0.317), trial (Figure 2A. *p* = 0.503, ηp^2^ = 0.158), or interactions (Figure 2A. *p* > 0.078, ηp^2^ < 0.436). RER was also lower when running downhill (Figure 2B. *p* = 0.007, ηp^2^ = 0.572), but there was a main effect of trial (Figure 2B. *p* < 0.001, ηp^2^ = 0.541), where the RER at trial 3 was lower than that at trial 1 *(p* < 0.001; *d =* 0.543) and trial 2 (*p* = 0.056; *d =* 0.423). There was no main effect of bout (Figure 2B. *p* = 0.817, ηp^2^ = 0.006) and no interactions (Figure 2B. *p* > 0.137, ηp^2^ < 0.224).

HR was lower when running downhill (Figure 2C. *p* = 0.005; ηp^2^ = 0.608), with no main effect of bout (Figure 2B. *p* = 0.178; ηp^2^ = 0.192), trial (Figure 2C. *p* = 0.843; ηp^2^ = 0.008), and no interactions (Figure 2C. *p* > 0.060; ηp^2^ < 0.268). Furthermore, BLa was lower when running downhill (Figure 2D. *p* = 0.015, ηp^2^ = 0.502) and was decreased in the second bout when compared to the first bout (Figure 2D. *p* = 0.005, ηp^2^ = 0.602). There was no main effect of trial (Figure 2D. *p* = 0.179, ηp^2^ = 0.174) and no interactions (Figure 2D. *p* > 0.134, ηp^2^ < 0.201).

For RPE, there was a Gradient*Trial (Figure 2E. *p* < 0.001; ηp^2^ = 0.847) and a Bout*Trial (Figure 2E. *p* = 0.007; ηp^2^ = 0.707) interaction, which were subsequently explored with pairwise comparisons. These tests indicated that, at trial 1, RPE was higher when running downhill compared to level (*p* = 0.051; *d =* 0.447). However, when gradient was compared at trial 2 (*p* = 0.003; *d =* 0.746) and trial 3 (*p* < 0.001; *d =* 1.325), RPE was higher for level running compared to downhill. Pairwise comparisons further indicated that, during level running, RPE increased after each trial (*p* < 0.020; *d >* 1.683). During the completion of the second bout, RPE was lower in first trial compared to that in the first bout (*p* = 0.002; *d =* 0.923), and only during the second bout, RPE was lower after trial 1 compared to that at trial 2 (*p* = 0.001; *d =* 1.338) and 3 (*p* = 0.01; *d =* 1.788).

### 3.2. Perception of Soreness

**Figure 3:**
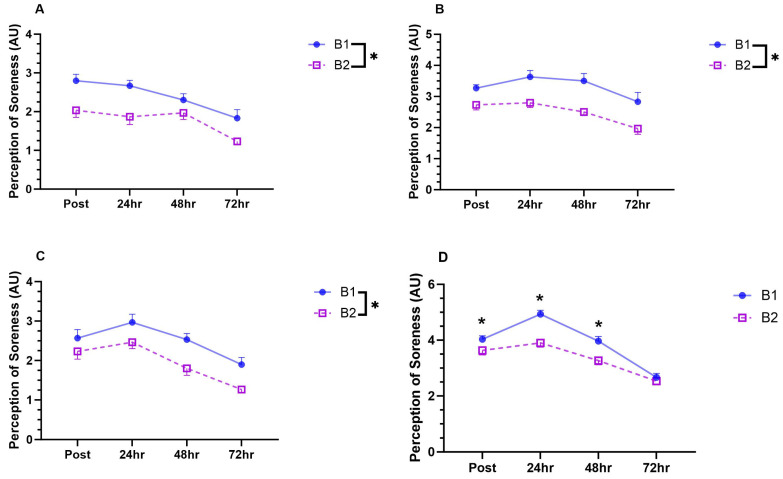
The effect of repeated bouts of downhill and level running on perceived post exercise soreness of the gastrocnemius (**A**), quadriceps (**B**), hamstrings (**C**), and gluteal muscles (**D**) [Data represented as mean ± standard error of mean (SEM); n = 10; B = Bout; Figures (**A**–**C**) * represent *p* < 0.05 for ANOVA main effect of bout; Figure (**D**) * represents *p* < 0.05 for between bout differences determined by pairwise comparisons].

The perception of soreness for the gastrocnemius was lower following completion of the second bout (Figure 3A. *p* = 0.001; ηp^2^ = 0.711), and there was a main effect of time (Figure 3A. *p* < 0.001; ηp^2^ = 0.592). Pairwise comparisons indicated that perceived soreness 72 h after was lower than that at all other measured time points (*p* < 0.022; *d >* 1.086). There was no Bout*Time interaction (Figure 3A. *p* = 0.276; ηp^2^ = 0.131). Similarly, the perception of soreness for the quadriceps was lower following completion of the second bout (Figure 3B. *p* = 0.001; ηp^2^ = 0.710), and there was a main effect of time (Figure 3B. *p* = 0.032; ηp^2^ = 0.695). Pairwise comparisons indicated that perceived soreness 72 h after was lower than that at all other measured time points (*p* < 0.033: *d >* 0.741). There was no Bout*Time interaction (Figure 3B. *p* = 0.508; ηp^2^ = 0.268).

The perception of soreness for the hamstrings was lower following completion of the second bout (Figure 3C. *p* = 0.001; ηp^2^ = 0.694), and there was a main effect of time (Figure 3C. *p* < 0.001; ηp^2^ = 0.941). Pairwise comparisons indicated perceived soreness 48 h after was reduced compared to and 24 h after (*p* = 0.013; *d >* 0.896), and soreness at 72 h after was lower than that at all other measured time points (*p* < 0.009: *d >* 1.200). There was no Bout*Time interaction (Figure 3C. *p* = 0.688; ηp^2^ = 0.052).

For the perception of soreness of the gluteal muscles, there was a Bout*Time interaction (Figure 3D. *p* = 0.016; ηp^2^ = 0.402). Subsequent pairwise comparisons indicated that perceived soreness was lower after the second bout when compared to the first bout immediately post exercise (*p* = 0.024: *d =* 0.923), 24 h post exercise (*p* < 0.001: *d =* 2.388), and 48 h post exercise (*p* = 0.021: *d =* 1.476). Upon completion of the first bout, the perception of soreness was higher 24 h post exercise compared to immediately after (*p* = 0.013: *d =* 2.262). The perception of soreness 72 h post exercise was lower than at all measured time points (*p* < 0.003: *d >* 2.795). Upon completion of the second bout, the perception of pain was lower 48 h after compared to at 24 h after (*p* = 0.027: *d >* 1.422), and was reduced 72 h after compared to all other time points (*p* < 0.002: *d >* 1.929).

## 4. Discussion

The present study evaluated the effect of repeated bouts of level and downhill running on physiological markers of effort and exercise induced muscle soreness in trained female distance runners. Irrespective of bout, downhill running resulted in reduced V̇O_2_, HR, BLa, and RER compared to level running. V̇O_2_, HR, and RER were not different in the second bout, refuting the hypothesis, however, V̇O_2_ was consistently lower in the downhill trials of the second bout. BLa was also lower in the second bout and RPE was lower in the first part of the trial. The first bout was effective in attenuating the perceived muscle soreness of the gastrocnemius, quadriceps, hamstrings, and gluteal muscles following the second bout, supporting the hypothesis. These data advance the current understanding of the RBE elicited by downhill running. Although the first bout of exercise had no effect on HR and RER, reduced BLa, perception of effort, perceived muscle soreness, and a trend for a reduced V̇O_2_ in the downhill trials during the second bout may be beneficial for distance running performance and highlights the potential benefits of an acute bout of downhill running as an effective conditioning intervention.

This study offers important new insight into the RBE that can be elicited from this mode of exercise. Previous evidence has focused on the influence of the RBE on attenuating impaired running economy in the days following a bout of downhill running [11,25], and only Khassetarash et al. [11] examined the effects of a repeated bout of downhill running on the energetic cost of 5 min of level running immediately following the damaging exercise bout. In the population of recreational male runners examined, V̇O_2_ and the energetic cost of running were unaffected, whilst V̇E was decreased. For the first time, the results of the present study demonstrate that the RBE elicited from the first trial did not affect V̇O_2_, HR, and RER when evaluated across multiple trials of both downhill and level running. However, it is noteworthy that, although the critical level of alpha was not reached in the ANOVA conducted, the effect size was large (*p* = 0.071, ηp^2^ = 0.317). Further investigation indicates that V̇O_2_ was lower at each of the downhill trials (Δ = 1.6–2.2 mL·kg^−1^·min^−1^) in the second bout at a level that represents a small effect (*d* = 0.382–0.426), as determined by Cohen’s *d* effect size calculations. Such effects are similar in magnitude to the benefits elicited by highly cushioned carbon plated ‘super’ racing shoes during level running [26] and, thus, could result in a meaningful improvement in distance running performance. Further to this, these data are the first to show that RBE from downhill running reduced BLa measured across repeated trials of level and negative gradient running. Such effects may be important to improve distance running performance over exercise durations or intensities greater than those examined in the present study, given that previous work indicated even moderate differences in initial BLa (from 3–5 mmol/L^−1^) and substantially reduced running economy in trained athletes [27]. However, future work should consider the translation of this directly to competitive distance running or time trial performance.

Mechanistically, the RBE has been shown to cause a shift toward the preferential recruitment of low-threshold motor units and improved motor unit synchronisation [15], which may act to reduce the oxygen deficit at the onset of steady state running. Indeed, a linear correlation has been previously demonstrated between oxygen deficit and BLa [28]. Furthermore, achieving the required level of muscular work through the duration of the running protocol via the greater activation of more oxidative lower-threshold motor units may reduce the demand on more glycolytic fibres, contributing to the reduced BLa. Such changes may not manifest in large or apparent changes in V̇O_2_, due to a greater number of active motor units to account for the reduced relative force output of slow twitch muscle fibres. The small changes in V̇O_2_ seen in the second bout of downhill running trials may be further explained by changes in gait biomechanics, where a subsequent bout of downhill running was shown to reduce the loss of lower limb stiffness and reduce energy absorption and generation on ground contact, resulting in a reduced energy expenditure to accomplish the same downhill running task [22]. Furthermore, RBE-induced changes in motor unit recruitment may be more apparent in eccentric contractions, thus, effects when running downhill may be more apparent. At present, caution is needed in the interpretation of proposed mechanisms, and future work is needed to enable a more precise understanding.

The reduced metabolic demand of downhill running compared to level running was expected, given that, at negative gradients, there is reduced muscular work for the generation of propulsion and an increase in metabolically efficient eccentric muscle actions needed for increased breaking [7]. Indeed, as per the results of the present work, several studies have demonstrated that, when compared to level running, downhill running results in reduced V̇O_2_, RER, HR, and metabolic power [4,5,6]. Given the increased contribution of eccentric muscle actions, acute and unaccustomed downhill running has been consistently shown to evoke exercise-induced muscle damage [1]. This was evident in the present study, given the elevated post exercise soreness of lower limb musculature that began to subside 48–72 h following each bout. Previous work indicates that downhill running elicits an increased perception of muscle soreness, in combination with increased blood markers of muscle of damage such as serum creatine kinase, markers of oxidative stress and myoglobin, and reduced maximal voluntary muscle function of the lower limb musculature [9,20,29,30]. Previous studies typically employ downhill running protocols of 30 min or greater, and there is evidence to support more severe effects in those that are less accustomed to this mode of exercise [1]. Interestingly, the results of the present study reveal that exercise-induced muscle damage still occurs in reasonably short trials of downhill running in a population that although did not specifically train on negative gradients, would likely not be completely naïve to running downhill.

The results of the present study further indicate that the initial bout of downhill running was protective against exercise-induced muscle damage, as evidenced by the reduction in the perceived soreness of the lower limb muscles in the days following the repeated bout. The protective effect of an initial bout of unaccustomed eccentric activity is the most widely explored phenomenon related to the RBE and attenuated muscle damage following a second bout of downhill running, which has been previously reported [1]. More specifically, previous work has demonstrated that muscle soreness, blood markers of muscle damage, the loss of maximal voluntary force of lower limb muscles, and the impact on post exercise running economy were reduced following a repeated bout of downhill running [9,10,11]. Our results demonstrate that attenuated muscle damage may occur after short bouts of downhill running, with benefits prevalent for trained athletes. Such effects may be valuable to distance runners by aiding post exercise recovery following intense exercise bouts, although work is needed to understand the time course over which such effects are prevalent and the transferability from other exercise modalities that can be used to elicit a RBE. Attenuated damage elicited by the RBE has been attributed to increased tendon compliance, the remodelling of skeletal muscles extracellular matrix, altered inflammatory responses, and increased motor unit synchronisation and recruitment, resulting in the redistribution of the workload across the muscle fibres [15]. Such effects may offer broader benefits to distance runners and team sport athletes, promoting advanced recovery between training and competitions.

### Limitations and Future Directions

Whilst this study offers important insight into the RBE elicited by downhill running, it is not without limitation. Although the number of participants is representative of the sample size used in previous studies [9,11,19,22], future work is needed to examine these effects in a larger and more diverse population of athletes. The treadmill protocol used to examine the RBE alternated between level and a negative gradient running. Though it is not possible to rule out some influence of level running on the RBE effects shown, participants were experienced distance runners, who were familiarised with level treadmill running, therefore, the between bout-differences in the measured outcomes are likely specific to the RBE elicited from downhill running. The study would have benefited from evaluation of gait biomechanics and neuromuscular assessment to understand further the mechanism underpinning the physiological data and the time course of recovery. However, such outcomes have been studied more extensively following downhill running and repeated bouts of eccentric exercise, allowing for sensible mechanistic justification of the results gained in the present work. Furthermore, several factors moderate the RBE, mainly, previous exposure to eccentric muscle activity, sex, and the specific mechanical exposure to eccentric actions (i.e., intensity, velocity, muscle length, number of actions, and duration between bouts) [15]. Therefore, these results may only be specific to the exercise protocol completed in the present study and the population assessed, and the influence of such factors should be the focus of future investigation. In particular, effects of a more severe bout of downhill running or an RBE evoked by other exercise modes (e.g., heavy-load eccentric resistance exercise) may elicit more pronounced adaptation and, subsequently, a greater performance-enhancing benefit during the second bout. However, there is a need to pragmatically balance the potential benefits of the RBE, with deleterious effects in the days following the initial exposure.

In line with this, damage induced from the exercise protocol and attenuation following the repeated bout was assessed by the perception of soreness and would have benefited from objective measures of damage and recovery, such as characterisation of neuromuscular performance, muscle morphology, and biomarkers of muscle damage. However, the wealth of data specific to damage induced by eccentric muscle actions indicate that perceived soreness well reflects objective measures of muscle damage [11,20]. In the context of the conditioning potential of RBE following exposure to downhill running, consideration of biomechanical and neuromuscular factors underpinning running performance would provide a useful avenue for future investigation. Such information would provide mechanistic insight that might account for the demonstrated effects, and if monitored in the post exercise period, would aid in devising appropriate recovery strategy.

Concurrent with advancing the understanding of the dose–response effect of downhill running on the RBE, future work should also consider evaluating longitudinally the time course of adaptation. For example, Nosaka et al. [16] demonstrated a faster recovery of muscle function and attenuation of soreness after 6 months that was not apparent when assessed at 9 and 18 months. However, this study was specific to localised eccentric exercise of the upper body using resistance exercise and, therefore, may not translate to the RBE effects elicited by downhill running. Furthermore, evidence suggests that the muscle groups of the arm are more susceptible to damage than lower limb muscles [31]. As such, understanding the prevalence of the RBE elicited from downhill running over durations longer than three weeks represents an important avenue for future investigation to guide eccentric exercise prescription.

## 5. Conclusions

In trained female distance runners, an initial bout of level and downhill running had no effect on HR and RER measured in a subsequent repeated bout. Although statistical analysis indicated that V̇O_2_ was also unaffected, estimates of effect size indicated a small reduction in V̇O_2_ at each of the downhill trials (Δ = 1.6–2.2 mL·kg^−1^·min^−1^) in the second bout. Furthermore, in the second bout BLa was lower and RPE was lower in the first part of the trial. The first bout was effective in attenuating perceived muscle soreness of lower limb musculature. These data infer that an initial bout of downhill running may modulate the physiological and perceived physical demand of a repeated bout of level and downhill running. Although the magnitude of the effect may be small, such effects may be beneficial for improving distance running performance, however, future work should consider directly examining the crossover effects to running performance, the mechanisms underpinning the adaptations, and the optimisation of downhill running protocol to maximise beneficial RBE effects.

## Figures and Tables

**Table 1:** Participant characteristics.

Age (years)	24.4 ± 2.0
Height (cm)	164.7 ± 1.5
Body mass (kg)	56.2 ± 1.8
V̇O_2peak_ (mL kg^−1^·min^−1^)	52.9 ± 1.1
70% vV̇O_2peak_ (km·h^−1^)	8.8 ± 1.4
1500 m PB time (min)	5.1 ± 0.4
5 km PB time (min)	21.48 ± 1.4
10 km PB time (min)	44.43 ± 3.3

Data represented as mean ± standard error of mean (SEM); n = 10: 70% vVO_2peak_ = Velocity at 70% V̇O_2peak_; PB = Personal Best data obtained from https://thepowerof10.info (accessed 1 March 2023).

## Data Availability

The data presented in this study are available on request from the corresponding author. The data are not publicly available due to containing information that could compromise the privacy of research participants.

## References

[1] Bontemps B., Vercruyssen F., Gruet M., Louis J. (2020). Downhill running: What are the effects and how can we adapt? A narrative review. Sports Med..

[2] Hessel A.L., Lindstedt S.L., Nishikawa K.C. (2017). Physiological mechanisms of eccentric contraction and its applications: A role for the giant titin protein. Front. Physiol..

[3] Proske U., Morgan D.L. (2001). Muscle damage from eccentric exercise: Mechanism, mechanical signs, adaptation and clinical applications. J. Physiol..

[4] Lemire M., Falbriard M., Aminian K., Millet G.P., Meyer F. (2021). Level, uphill, and downhill running economy values are correlated except on steep slopes. Front. Physiol..

[5] Whiting C.S., Hoogkamer W., Kram R. (2022). Metabolic cost of level, uphill, and downhill running in highly cushioned shoes with carbon-fiber plates. J. Sport Health Sci..

[6] Robergs R.A., Wagner D.R., Skemp K.M. (1997). Oxygen consumption and energy expenditure of level versus downhill running. J. Sports Med. Phys. Fitness.

[7] Abbott B.C., Bigland B., Ritchie J.M. (1952). The physiological cost of negative work. J. Physiol..

[8] Vernillo G., Giandolini M., Edwards W.B., Morin J.B., Samozino P., Horvais N., Millet G.Y. (2017). Biomechanics and physiology of uphill and downhill running. Sports Med..

[9] Park K.S., Lee M.G. (2015). Effects of unaccustomed downhill running on muscle damage, oxidative stress, and leukocyte apoptosis. J. Exerc. Nutr. Biochem..

[10] McKune A.J., Smith L.L., Semple S.J., Mokethwa B., Wadee A.A. (2006). Immunoglobulin responses to a repeated bout of downhill running. Br. J. Sports Med..

[11] Khassetarash A., Vernillo G., Krüger R.L., Edwards W.B., Millet G.Y. (2022). Neuromuscular, biomechanical, and energetic adjustments following repeated bouts of downhill running. J. Sport Health Sci..

[12] Southall-Edwards R., Innes S., Ali A., Jones B. (2020). The effect of downhill running conditions on muscle damage in recreationally active adults. J. Hum. Sport Exerc..

[13] Kohne J.L., Ormsbee M.J., McKune A.J. (2016). The effects of a multi-ingredient supplement on markers of muscle damage and inflammation following downhill running in females. J. Int. Soc. Sports Nutr..

[14] Braun W.A., Dutto D.J. (2003). The effects of a single bout of downhill running and ensuing delayed onset of muscle soreness on running economy performed 48 h later. Eur. J. Appl. Physiol..

[15] Hyldahl R.D., Chen T.C., Nosaka K. (2017). Mechanisms and mediators of the skeletal muscle repeated bout effect. Exerc. Sport Sci. Rev..

[16] Nosaka K., Sakamoto K., Newton M., Sacco P. (2001). How long does the protective effect on eccentric exercise-induced muscle damage last?. Med. Sci. Sports Exerc..

[17] McKay A.K.A., Stellingwerff T., Smith E.S., Martin D.T., Mujika I., Goosey-Tolfrey V.L., Sheppard J., Burke L.M. (2022). Defining training and performance caliber: A participant classification framework. Int. J. Sports Physiol. Perform..

[18] Vernillo G., Aguiar M., Savoldelli A., Martinez A., Giandolini M., Horvais N., Edwards W.B., Millet G.Y. (2020). Regular changes in foot strike pattern during prolonged downhill running do not influence neuromuscular, energetics, or biomechanical parameters. Eur. J. Sport Sci..

[19] Chen T.C., Nosaka K., Tu J.H. (2007). Changes in running economy following downhill running. J. Sports Sci..

[20] De Oliveira Assumpção C., Barreto R.V., de Lima L.C.R., Cardozo A.C., de Lima Montebelo M.I., Catarino H.R.C., Greco C.C., Denadai B.S. (2020). A single bout of downhill running attenuates subsequent level running-induced fatigue. Sci. Rep..

[21] Talag T.S. (1973). Residual muscular soreness as influenced by concentric, eccentric, and static contractions. Res. Q..

[22] Khassetarash A., Baggaley M., Vernillo G., Millet G.Y., Edwards W.B. (2023). The repeated bout effect influences lower-extremity biomechanics during a 30-min downhill run. Eur. J. Sport Sci..

[23] Cohen J. (1988). Statistical Power Analysis for the Behavioral Sciences.

[24] Hopkins W.G., Marshall S.W., Batterham A.M., Hanin J. (2009). Progressive statistics for studies in sports medicine and exercise science. Med. Sci. Sports Exerc..

[25] Chen T.C., Chen H.-L., Wu C.-J., Lin M.-R., Chen C.-H., Wang L.-I., Wang S.-Y., Tu J.-H. (2007). Changes in running economy following a repeated bout of downhill running. J. Exerc. Sci. Fit..

[26] Joubert D.P., Jones G.P. (2022). A comparison of running economy across seven highly cushioned racing shoes with carbon-fibre plates. Footwear Sci..

[27] Hoff J., Støren Ø., Finstad A., Wang E., Helgerud J. (2016). Increased blood lactate level deteriorates running economy in world class endurance athletes. J. Strength Cond. Res..

[28] Gildein H.P., Kaufmehl K., Last M., Leititis J., Wildberg A., Mocellin R. (1993). Oxygen deficit and blood lactate in prepubertal boys during exercise above the anaerobic threshold. Eur. J. Pediatr..

[29] Byrnes W.C., Clarkson P.M., White J.S., Hsieh S.S., Frykman P.N., Maughan R.J. (1985). Delayed onset muscle soreness following repeated bouts of downhill running. J. Appl. Physiol..

[30] Varesco G., Coratella G., Rozand V., Cuinet B., Lombardi G., Mourot L., Vernillo G. (2022). Downhill running affects the late but not the early phase of the rate of force development. Eur. J. Appl. Physiol..

[31] Jamurtas A.Z., Theocharis V., Tofas T., Tsiokanos A., Yfanti C., Paschalis V., Koutedakis Y., Nosaka K. (2005). Comparison between leg and arm eccentric exercises of the same relative intensity on indices of muscle damage. Eur. J. Appl. Physiol..

